# Methylenetetrahydrofolate reductase polymorphisms and interaction with smoking and alcohol consumption in lung cancer risk: a case-control study in a Japanese population

**DOI:** 10.1186/1471-2407-11-459

**Published:** 2011-10-25

**Authors:** Chikako Kiyohara, Takahiko Horiuchi, Koichi Takayama, Yoichi Nakanishi

**Affiliations:** 1 Department of Preventive Medicine, Graduate School of Medical Sciences, Kyushu University, Maidashi 3-1-1, Higashi-ku, Fukuoka 812-8582, Japan; 2 Department of Medicine and Biosystemic Science, Graduate School of Medical Sciences, Kyushu University, Fukuoka 812-8582, Japan; 3 Research Institute for Diseases of the Chest, Graduate School of Medical Sciences, Kyushu University, 3-1-1 Maidashi, Higashi-ku, Fukuoka 812-8582, Japan

## Abstract

**Background:**

Cigarette smoking is an established risk factor of lung cancer development while the current epidemiological evidence is suggestive of an increased lung cancer risk associated with alcohol consumption. Dietary folate, which is present in a wide range of fresh fruits and vegetables, may be a micronutrient that has a beneficial impact on lung carcinogenesis. Methylenetetrahydrofolate reductase (MTHFR) plays a crucial role in regulating folate metabolism, which affects both DNA synthesis/repair and methylation. We examined if smoking or alcohol consumption modify associations between *MTHFR *polymorphisms and lung cancer risk.

**Methods:**

We evaluated the role of the *MTHFR *C677T (rs1801133) and A1298C (rs1801131) polymorphisms in a case-control study comprised of 462 lung cancer cases and 379 controls in a Japanese population. Logistic regression was used to assess the adjusted odds ratios (OR) and 95% confidence intervals (95% CI).

**Results:**

The TT genotype of the C677T polymorphism was significantly associated with an increased risk of lung cancer (OR = 2.27, 95% CI = 1.42 - 3.62, P < 0.01) while the A1298C polymorphism was not associated with lung cancer risk. The minor alleles of both polymorphisms behaved in a recessive fashion. The highest risks were seen for 677TT-carriers with a history of smoking or excessive drinking (OR = 6.16, 95% CI = 3.48 - 10.9 for smoking; OR = 3.09, 95% CI = 1.64 - 5.81 for drinking) compared with C-carriers without a history of smoking or excessive drinking, but no interactions were seen. The 1298CC genotype was only associated with increased risk among non-smokers (P < 0.05), and smoking was only associated with increased risks among 1298A-carriers (P < 0.01), but no significant interaction was seen. There was a synergistic interaction between the A1298C polymorphism and drinking (P < 0.05). The highest risk was seen for the CC-carriers with excessive drinking (OR = 7.24, 95% CI = 1.89 - 27.7) compared with the A-carriers without excessive drinking).

**Conclusions:**

The C677T polymorphism was significantly associated with lung cancer risk. Although the A1298C polymorphism was not associated with lung cancer risk, a significant interaction with drinking was observed. Future studies incorporating data on folate intake may undoubtedly lead to a more thorough understanding of the role of the *MTHFR *polymorphisms in lung cancer development.

## Background

Lung cancer remains one of the major causes of mortality worldwide [1]. Although cigarette smoking is the primary risk factor for lung cancer, approximately one in 10 smokers develops lung cancer in their lifetime indicating an interindividual variation in susceptibility to tobacco smoke [2]. Other factors such as dietary factors may also play an important role in the etiology of lung cancer. Convincing evidence shows an inverse association between fruit and vegetable intake and lung cancer risk [3-5].

Genetic host factors have been implicated in some of the observed differences in susceptibility. To date, candidate susceptibility genes for lung cancer have been extensively studied, with most of the work focusing on mechanistically plausible polymorphisms in genes coding for enzymes involved in the activation, detoxification and repair of damage caused by tobacco smoke. In addition to metabolic polymorphisms, functional polymorphisms in folate metabolizing genes can also be good candidate susceptibility polymorphisms for lung cancer susceptibility. Folate, which is unsynthesizable by humans, is one of the major components of fruits and vegetables and may exert a beneficial impact on lung carcinogenesis [6]. Methylenetetrahydrofolate reductase (MTHFR), a key enzyme in folate metabolism, irreversibly catalyzes the conversion of 5,10-methylenetettrahydrofolate (5,10-methylene THF) to 5-methyltetrahydrofolate (5-methly THF). Two common functional *MTHFR *polymorphisms, C677T (rs1801133, A222V) and A1298C (rs1801131, E429A), have been the most studied. The TT genotype of the C677T polymorphism results in 30% enzyme activity *in vitro *compared with the CC genotype [7], whereas the CC genotype of the A1298C polymorphism has 60% enzyme activity of the AA genotype *in vitro *[8,9]. Individuals with the genotypes involved in reduced enzyme activity had significantly increased homocysteine levels and decreased folate levels compared with individuals with their counterpart genotypes [10]. The importance of the MTHFR enzyme in cancer susceptibility arises from its involvement in two pathways of folate metabolism. 5,10-methylene THF is required for DNA synthesis and DNA repair, and 5-methyl THF is the methyl donor for regeneration of methionine from homocysteine for subsequent methylation reactions [11,12]. Decrease in the activity of the MTHFR enzyme increases the pool of 5,10-methylene THF at the expense of the pool of 5-methyl THF (contributes to downstream methylation reactions by regeneration of methionine from homocysteine). Enhanced availability of 5,10-methylene THF in the DNA synthesis pathway reduces misincorporation of uracil into DNA, which might otherwise result in strand breaks during uracil excision repair, thus increasing the risk of chromosomal aberrations [11]. Therefore, it is probable that the decreased availability of 5-methyl THF for DNA methylation is the crucial mechanism behind the expected increased risk of lung cancer in subjects with the genotypes related to low MTHFR activity.

Lung cancer is a common disease that results from a complex interplay of genetic and environmental risk factors. It has been reported that there are lower circulating folate concentrations in smokers than in nonsmokers [13,14] although a considerable portion of the effect of cigarette smoking on folate concentrations may be indirect (different intake of smokers and nonsmokers). Alcohol consumption has been shown to reduce folate bioavailability [15]. Smokers (established high risk population) or drinkers (suspected high risk population) with a genotype associated with decreased folate levels may be more susceptible to lung cancer than expected from the independent effects of the two (smoking/drinking and genetic) separate factors. As smoking and drinking may interact with the *MTHFR *genotypes to induce lung carcinogenesis, we conducted a case-control study of lung cancer in a Japanese population with special attention to the interaction between the *MTHFR *polymorphisms and either cigarette smoking or alcohol drinking.

## Methods

### Study subjects and data collection

Lung cancer patients were recruited at Kyushu University Hospital (Research Institute for Diseases of the Chest, Kyushu University) and its collaborating hospitals. Eligible cases were newly diagnosed and histologically confirmed primary lung cancer during the period from 1996 to 2008. Histological types were categorized into four major types according to the International Classification of Diseases for Oncology (ICD-O), second edition: adenocarcinoma (8140, 8211, 8230-8231, 8250-8260, 8323, 8480-8490, 8550-8560, 8570-8572), squamous cell carcinoma (8050-8076), small cell carcinoma (8040-8045) and large cell carcinoma (8012-8031, 8310). The participation rate among the cases was 100%. Three hundred and seventy nine potential controls were selected from inpatients without having a clinical history of any type of cancer, past or present, ischemic heart disease and chronic respiratory diseases who stayed in departments other than departments of respiratory medicine, such as the department of internal medicine, in the collaborating hospitals during the same period because hospital controls are more motivated and are more easily accessible for obtaining DNA samples. Controls were not, individually or in larger groups, matched to cases. Controls were approached by their attending physicians to be recruited as control subjects. None of the controls refused to participate in this study. A self-administered questionnaire was used to collect data on demographic and lifestyle factors such as age, years of education, smoking, alcohol consumption, environmental tobacco exposure from spouse and so on. All subjects were unrelated ethnic Japanese.

The study protocol was approved by our institutional review board, and all participants provided written informed consent.

### Genetic analyses

Genomic DNA was extracted from blood samples. Genotyping was conducted with blinding to case/control status. The genotyping of the C677T polymorphism was performed with TaqMan assay (genotyping protocols supplied centrally by IARC because this single nucleotide polymorphism (SNP) genotyping is part of an IARC-oriented international collaborative study on lung cancer) while the A1298C genotypes were evaluated independently of the IARC-oriented international collaborative study on lung cancer using the PCR-restriction fragment length polymorphism (RFLP) method described elsewhere [8]. Generally, the concordance rate between PCR-RFLP genotyping and real-time PCR assay is high [16]. For quality control, both assays were repeated on a random 5% of all samples, and the replicates were 100% concordant.

### Statistical analysis

To test for associations between SNPs and lung cancer, we defined the ancestral allele using the National Center for Biotechnology Information SNP database as the major allele. We assessed HWE via a goodness-of-fit χ^2 ^test (Pearson) to compare the observed and expected genotype frequencies among controls. Based on the results from functional studies and the associations between *MTHFR *polymorphisms and lung cancer, we designated the genotype that is presumed to increase the risk of lung cancer as the "at-risk" genotype. The trend of association was assessed by a logistic regression model assigning ordinal scores to the levels of the independent variable. Unconditional logistic regression was used to compute the odds ratios (ORs) and their 95% confidence intervals (CIs), with adjustments for several covariates found to be associated with risk (age, sex, smoking status and education). Subjects were considered current smokers if they had smoked or stopped smoking less than one year before either the date of diagnosis (lung cancer patients) or the date of completion of the questionnaires (controls). Non-smokers were defined as those who had never smoked in their lifetime. Former smokers were those who had stopped smoking one or more years before either the date of diagnosis of lung cancer (lung cancer patients) or the date of completion of the questionnaires (controls). Based on "Healthy Japan 21" (National Health Promotion in the 21st Century), heavy drinkers were defined as those who drank more than 60 g per day of alcohol [17]. As "Healthy Japan 21" has emphasized drinking an appropriate volume of alcohol (20 g of alcohol per day), appropriate drinkers were defined as those who did not exceed 20 g of alcohol intake per day. The appropriate volume of alcohol use may have a protective effect on life expectancy and morbidity [18]. Moderate drinkers were defined as those who drank alcohol more than 20 g per day but not exceeding 60 g per day. Unlike cigarette smoking, ingested alcohol is eliminated from the body by various metabolic mechanisms, and the alcohol elimination process begins almost immediately. Significant relationships between excessive drinking and lung cancer have been reported while appropriate drinking has not shown the same effects [19]. In terms of alcohol consumption, the subjects were classified into the following two groups based on their intake for at least one year as follows: those who drink more than 20 g of alcohol per day (excessive drinkers) and those who drink less than 20 g of alcohol per day (appropriate drinkers). The interaction between *MTHFR *polymorphisms and smoking/drinking on the risk of lung cancer was statistically evaluated based on the likelihood test, comparing the models with and without (multiplicative scale) terms for interaction. We used two logistic regression models to specifically test for dominant versus recessive inheritance of the effects of the T allele. In the dominant model, we hypothesized that the *MTHFR *C677T (A1298C) genotypes CT (AC) and TT (CC) contributed equally to lung risk, and we coded CC (AA) = 0, CT (AC) = 1 and TT (CC) = 1. In the recessive model, we hypothesized that only the TT (CC) genotype contributed to the risk of the disease, and we coded CC (AA) = 0, CT (AC) = 0 and TT (C) = 1. The log-likelihood statistics of each model were compared with the general logistic regression log likelihood that jointly fitted both dominant and recessive effects. Linkage disequilibrium between the C677T and A1298C polymorphisms was calculated with HaploView software version 4.2 [20].

All statistical analyses were performed using the computer program STATA Version 10.1 (STATA Corporation, College Station, TX). All *P *values were two-sided, with those less than 0.05 considered statistically significant.

## Results

The distributions of selected characteristics among study subjects are summarized in Table 1. Our analysis included 462 lung cancer patients (242 with adenocarcinoma, 131 with squamous cell carcinoma, 69 with small cell carcinoma, and 20 with large cell carcinoma). As controls were not selected to match lung cancer patients on age and sex, there was a significant difference in age (P < 0.01) and sex ratio (P < 0.01) between lung cancer patients and controls. Similarly, there were significant differences between cases and controls in terms of sex ratio, smoking status, pack-years of smoking and years of education.

**Table 1:** Selected characteristics of lung cancer cases and controls

Characteristics	Cases (n = 462)	Controls (n = 379)	P
Age (year), median (IQR)	68 (62 - 73)	58 (48 - 65)	<0.01
Male, n (%)	287 (62.1)	283 (74.7)	<0.01
Smoking status, n (%)			<0.01
Current smoker	198 (42.9)	129 (34.0)	
Former smoker	111 (24.0)	41 (10.8)	
Never smoker	153 (33.1)	209 (55.2)	
Pack years, median (IQR)	38 (0 - 58)	0 (0 - 34)	<0.01
Moderate/heavy drinkers, n (%)	284 (61.5)	175 (46.2)	<0.01
Exposure to environmental tobacco smoke among non-smokers, n (%)	99 (64.7)	135 (64.6)	0.98
Education, median (IQR)	12 (12 - 16)	16 (12 - 16)	<0.01
Histology, n (%)			
Adenocarcinoma	242 (52.4)		
Squamous cell carcinoma	131 (28.4)		
Small cell carcinoma	69 (14.9)		
Large cell carcinoma	20 (4.3)		

IQR, interquartile range

As shown in Table 2, the frequencies of the CC (ancestral based on National Center for Biotechnology Information SNP database), CT and TT genotypes of the *MTHFR *C677T polymorphism were 33.1%, 43.5% and 23.4% in cases and 41.7%, 44.9% and 13.5% in controls, respectively. The genotype distribution of the C677T polymorphism was consistent with HWE among controls (P_HWE _= 0.62). The distributions of the AA (ancestral), AC and CC genotypes of the A1298 polymorphism were 60.2%, 33.3% and 6.49% in cases and 63.1%, 32.2% and 4.75% in controls, respectively. This polymorphism was also in HWE among controls (P_HWE _= 0.63). The TT genotype of the C677T polymorphism was significantly associated with an increased risk of lung cancer compared with the CC genotype (adjusted OR = 2.27, 95% CI = 1.42 - 3.63, P < 0.01). With the CC genotype as reference, the OR for the combined TT and CT genotypes was 1.49 (95% CI = 1.08 - 2.07, P = 0.02) (dominant model). Comparing the dominant model with the general model, the χ^2 ^value was 6.37 (P = 0.01), so the dominant model was rejected. Using the CC and CT genotypes combined as the reference, the OR for the TT genotype was 2.00 (95% CI = 1.30 - 3.07, P < 0.01), and the χ^2 ^value of the recessive model was 1.81 (P = 0.18) against the general model. These findings suggest a recessive effect of the T allele on lung cancer risk. On the other hand, the association of rs18001131 with lung cancer was not significant in the general, dominant and recessive models. The χ^2 ^value of the dominant model was 1.60 (P = 0.21) against the general model while that of the recessive model was 0.041 (P = 0.84). These findings suggest that the recessive model fits better than the dominant model. Based on the results of the likelihood ratio test, subjects with at least one ancestral allele were bundled in one group for subsequent analysis. A high degree of linkage disequilibrium was observed between the C677T and A1298C polymorphisms (D' = 0.93, r^2 ^= 0.26; data not shown).

**Table 2:** Association between the *MTHFR *polymorphisms and risk of lung cancer

	Number (%) of			OR (95% CI)			
			
Polymorphism	Cases	Controls	P_HWE_^†^	Crude	P	Adjusted*	P
C677T							
CC (ancestral**)	153 (33.1)	158 (41.7)		1.0 (reference)	-	1.0 (reference)	-
CT	201 (43.5)	170 (44.9)		1.22 (0.90 - 1.65)	0.20	1.27 (0.90 - 1.80)	0.18
TT	108 (23.4)	51 (13.5)	0.62	2.19 (1.47 - 3.26)	<0.01	2.27 (1.42 - 3.62)	<0.01
Dominant model CT + TT vs.CC				1.44 (1.09 - 1.91)	0.01	1.49 (1.08 - 2.07)	0.02
Recessive model TT vs. CT + CC				1.96 (1.36 - 2.83)	<0.01	2.00 (1.30 - 3.07)	<0.01
							
A1298C							
AA (ancestral**)	278 (60.2)	239 (63.1)		1.0 (reference)	-	1.0 (reference)	-
AC	154 (33.3)	122 (32.2)		1.08 (0.81 - 1.46)	0.59	0.97 (0.69 - 1.35)	0.84
CC	30 (6.49)	18 (4.75)	0.63	1.43 (0.78 - 2.64)	0.25	1.55 (0.76 - 3.17)	0.23
Dominant model AC + CC vs. AA				1.13 (0.85 - 1.49)	0.39	1.03 (0.75 - 1.42)	0.85
Recessive model CC vs. AC + AA				1.39 (0.76 - 2.54)	0.28	1.57 (0.77 - 3.18)	0.21

* Adjusted for age, sex, education, smoking status and drinking.

** Defined by National Center for Biotechnology Information SNP database.

^† ^P for Hardy-Weinberg equilibrium test among controls.

Table 3 shows the modifying effect of the C677T genotypes on the association of smoking or drinking with lung cancer risk. To achieve adequate statistical power, current and former smokers were combined (ever-smokers). A history of smoking (adjusted OR = 3.17; 95% CI = 2.28 - 4.40, P < 0.01) and excessive drinking (adjusted OR = 1.76; 95% CI = 1.27 - 2.43, P < 0.01) were associated with an increased risk of lung cancer (data not shown). Smokers with the TT genotype ("at-risk" genotype) (adjusted OR = 6.16, 95% CI = 3.48 - 10.9, P < 0.01) had a significantly higher risk of lung cancer than non-smokers with at least one C allele (reference). The "at-risk" genotype was associated with an increased risk of lung cancer in both non-smokers (OR = 1.95, 95% CI = 0.99 - 3.84, P = 0.053) and ever-smokers (OR = 1.97, 95% CI = 1.14 - 3.12, P = 0.015, data not shown). Ever-smoking was associated with an increased risk of lung cancer in both subjects with at least one C allele (OR = 3.03, 95% CI = 2.12 - 4.34, P < 0.01) and those with the "at-risk" genotype (OR = 3.17, 95% CI = 1.35 -7.41, P < 0.01, data not shown). The multiplicative interaction between the C677T genotypes and smoking was far from significant (P = 0.93). Similarly, excessive drinkers with the "at-risk" genotype (adjusted OR = 3.09, 95% CI = 1.64 - 5.81, P < 0.01) had a higher risk of lung cancer than appropriate drinkers with at least one C allele (reference). The "at-risk" genotype was associated with an increased risk of lung cancer in both appropriate drinkers (OR = 2.46, 95% CI = 1.37 - 4.43, P < 0.01) and excessive drinkers (OR = 1.58, 95% CI = 0.86 - 2.89, P = 0.14, data not shown). Excessive drinking was significantly associated with an increased risk of lung cancer in subjects with at least one C allele (OR = 1.97, 95% CI = 1.38 -2.82, P < 0.01) but not in those with the "at-risk" genotype (OR = 1.22, 95% CI = 0.52 - 2.82, P = 0.65, data not shown). No evidence of interaction between the C667T polymorphism and drinking was detected (P_interaction _= 0.30).

**Table 3:** Interaction of the *MTHFR *C677T polymorphism and cigarette smoking or alcohol drinking

	Non-smokers					Ever-smokers				
	
Genotype	Cases/	OR (95% CI)				Cases/	OR (95% CI)			
		
	Controls	Crude	P	Adjusted*	P	Controls	Crude	P	Adjusted*	P
CC + CT	126/181	1.0 (reference)	-	1.0 (reference)	-	228/147	2.23 (1.64 - 3.03)	<0.01	3.03 (2.12 - 4.34)	<0.01
TT	27/28	1.39 (0.78 - 2.46)	0.28	1.95 (0.99 - 3.84)	0.05**	81/23	5.06 (3.02 - 8.47)	<0.01	6.16 (3.48 - 10.9)	<0.01
Crude P_interaction _= 0.21 and adjusted P_interaction _= 0.93										

	Appropriate drinkers				Excessive drinkers					
	
Genotype	Cases/	OR (95% CI)				Cases/	OR (95% CI)			
		
	Controls	Crude	P	Adjusted*	P	Controls	Crude	P	Adjusted*	P

CC + CT	128/177	1.0 (reference)	-	1.0 (reference)	-	226/151	2.07 (1.52 - 2.81)	<0.01	1.97 (1.38 - 2.82)	<0.01
TT	50/27	2.56 (1.52 - 4.31)	<0.01	2.46 (1.37 - 4.43)	<0.01	58/24	3.34 (1.97 - 5.66)	<0.01	3.09 (1.64 - 5.81)	<0.01
Crude P_interaction _= 0.22 and adjusted P_interaction _= 0.30										

* Adjusted for age, sex, education and smoking or drinking.

**Exact P = 0.053.

We assessed interactions between the A1298C polymorphism and smoking or drinking. As shown in Table 4, smokers with the CC genotype ("at-risk" genotype) (adjusted OR = 3.15, 95% CI = 1.24 - 7.98) had a significantly higher risk of lung cancer than non-smokers with at least one A allele (reference). The "at-risk" genotype was significantly associated with an increased risk of lung cancer in non-smokers (OR = 2.82, 95% CI = 1.02 - 7.83, P = 0.049) but not in ever-smokers (OR = 0.88, 95% CI = 0.35 - 2.02, P = 0.79, data not shown). Ever-smoking was significantly associated with an increased risk of lung cancer in subjects with at least one C allele (OR = 3.40, 95% CI = 2.42 -4.78, P < 0.01) but not in those with the "at-risk" genotype (OR = 0.86, 95% CI = 0.17 - 4.26, P = 0.86, data not shown). There was no significant interaction between the A1298C polymorphism and smoking (P_interaction _= 0.11). Excessive drinkers with the "at-risk" genotype (adjusted OR = 7.24, 95% CI = 1.89 - 27.7, P < 0.01) had a higher risk of lung cancer than those with at least one A allele (adjusted OR = 1.64, 95% CI = 1.18 - 2.29, P < 0.01), relative to appropriate drinkers with at least one A allele (reference). The "at-risk" genotype was significantly associated with an increased risk of lung cancer in excessive drinkers (OR = 3.96, 95% CI = 1.08 - 14.5, P < 0.01, data not shown) but not in appropriate drinkers (OR = 0.86, 95% CI = 0.35 - 2.16, P = 0.76). Excessive drinking was significantly associated with an increased risk of lung cancer in both subjects with at least one A allele (OR = 1.64, 95% CI = 1.18 - 2.29, P < 0.01) and those with the "at-risk" genotype (OR = 11.8, 95% CI = 1.81 - 76.5, P = 0.01, data not shown). The multiplicative (synergistic) interaction measure was statistically significant (P_interaction _= 0.049).

**Table 4:** Interaction of the *MTHFR *A1298C polymorphism and cigarette smoking or alcohol drinking

	Non-smokers					Ever-smokers				
	
Genotype	Cases/	OR (95% CI)				Cases/	OR (95% CI)			
		
	Controls	Crude	P	Adjusted*	P	Controls	Crude	P	Adjusted*	P
AA + AC	141/200	1.0 (reference)	-	1.0 (reference)	-	291/161	2.56 (1.92 - 3.42)	<0.01	3.40 (2.42 - 4.78)	<0.01
CC	12/9	1.89 (0.78 - 4.61)	0.16	2.82 (1.02 - 7.83)	0.05**	18/9	2.83 (1.23 - 6.50)	0.01	3.15 (1.24 - 7.98)	0.02
Crude P_interaction _= 0.39 and adjusted P_interaction _= 0.11										

	Appropriate drinkers					Excessive drinkers				
	
Genotype	Cases/	OR (95% CI)				Cases/	OR (95% CI)			
		
	Controls	Crude	P	Adjusted*	P	Controls	Crude	P	Adjusted*	P

AA + AC	167/190	1.0 (reference)	-	1.0 (reference)	-	265/171	1.76 (1.33 - 2.34)	<0.01	1.64 (1.18 - 2.29)	<0.01
CC	11/14	0.89 (0.40 - 2.02)	0.79	0.86 (0.35 - 2.16)	0.76	19/4	5.40 (1.80 - 16.2)	<0.01	7.24 (1.89 - 27.7)	<0.01
Crude P_interaction _= 0.08 and adjusted P_interaction _= 0.05†										

* Adjusted for age, sex, education and smoking or drinking.

**Exact P = 0.046 †Exact P = 0.049

## Discussion

The present study showed that the TT genotype of the *MTHFR *C677T polymorphism was significantly associated with an increased risk of lung cancer (OR = 2.27, 95% CI = 1.42 - 3.62, P < 0.01). Although the *MTHFR *A1298C polymorphism was not associated with lung cancer risk, there was a significant synergistic interaction between the A1298C polymorphism and alcohol consumption (P_interaciton _= 0.049).

Among controls, the prevalences of the C allele of the C677T polymorphism and the A allele of the A1298C polymorphism were 64.1% and 79.1%, respectively (data not shown). According to the HapMap SNP database [21], the C allele frequency of the C677T polymorphism is most common among Yorubas (a West African ethnic group, 89.7%) and least common among Han Chinese (48.9%); Japanese (63.3%) and Caucasians (76.3%) have intermediate frequencies. The frequency of the C allele in our study was similar to the HapMap SNP database. Meanwhile, the A allele frequencies of the A1298C polymorphism among Caucasians, Han Chinese, Japanese and Yorubas were 64.2%, 80.0%, 82.2% and 89.2%, respectively, according to the HapMap SNP database [22]. The frequency of the A allele in our study was somewhat lower than the HapMap SNP database but similar to other Japanese populations [23-25] (79.0%, 78.4% and 78.9%).

As shown in Table 2, the TT genotype of the *MTHFR *C677T polymorphism was significantly associated with an increased risk of lung cancer (OR = 2.27, 95% CI = 1.42 - 3.62). Results in terms of the association between the C677T polymorphism and lung cancer yielded mixed, variously reporting an increased risk [26-31], a decreased risk [25,32-35] or no association [36-39]. Two meta-analyses on the association between lung cancer and the *MTHFR *polymorphisms have been published in 2008 [40] and 2009 [41], respectively. The first meta-analysis [40] based on the published data from eight individual case-control studies [25-29,32,33,37] reported that the summary OR for the TT genotype was 1.12 (95% CI = 0.97 -1.28) compared with the CC genotype. The second meta-analysis [41] reported that the summary OR for the TT genotype was 1.37 (95% CI = 1.02 - 1.84) when excluding studies conducted in the USA, where some common food items are regularly fortified with folate since 1998 [42]. Differences in ethnicity, dietary intake, exposure to environmental carcinogens and sample size may be responsible for the discrepancies in the results. It has been suggested that cancer risk associated with the *MTHFR *polymorphisms may be modulated by folate intake [43,44]. Folate supplements (excessive) have been shown to increase folic acid levels, reduce homocysteine levels and then restore normal methionine levels, particularly in these individuals with the TT genotype of the *MTHFR *polymorphism [45,46] although the mechanism remains unclear. Normal methionine levels may not induce aberrant DNA methylation [43]. When folate is excess, the C677T polymorphism may not affect lung cancer risk. Since Japanese drink several cups of green tea daily and consume substantial vegetables and fruits [47], folate insufficiency is rare. As the prevalence of folate supplement users is very low in Japan (0.1%) [48], excessive folate intake is also rare. When folate intake is sufficient, individuals with the TT genotype of the C677T polymorphism may have an increased risk of lung cancer, because decrease in MTHFR activity might lead to impairment of DNA methylation due to a reduction in the availability of 5-methyl THF.

The A1298C polymorphism was not associated with lung cancer risk in the present study. The A1298C polymorphism, which has been much less examined, was not associated with lung cancer risk in this study. Although the C677T and A1298C polymorphisms are in strong linkage disequilibrium, the different impacts of the two polymorphisms may be expected due to their location within the protein and subsequent effect on function. The meta-analysis [40] of seven studies [25-29,33,37] also showed no association between lung cancer and the A1298C polymorphism. Two [33,38] of 10 studies [25-29,31,33,34,37,38] showed a significant deleterious effect of the CC genotype of the A1298C polymorphism on lung cancer. The lower prevalence of the C allele of the A1298C polymorphism may make researchers less likely to detect a significant association. It is difficult to estimate accurately the combined effects of the C677T and A1298C polymorphisms on lung cancer risk in the current sample size. The TT genotype of the C677T polymorphism has a greater impact on enzyme function [7-9]. Moreover, several *in vivo *studies demonstrated an association between the TT genotype of the C677T polymorphism and increased total homocysteine plasma levels in healthy subjects while limited and inconsistent data on the role of the A1298C polymorphism as determinant of total homocysteine plasma levels are available [49,50]. The C677T polymorphism may have confounded the results concerning the A1298C polymorphism. Further research on larger study populations is required before definitive conclusions can be drawn.

It is widely accepted that lung cancer development requires environmental factors acting on a genetically predisposed individual. Studying gene-environment interactions in relation to risk of lung cancer may be valuable because positive findings would clearly implicate the substrates with which the gene interacts as disease-causing exposures, clarify lung cancer etiology, and point to environmental modifications for disease prevention. We evaluated whether an interaction existed between cigarette smoking and the C677T or A1298C polymorphism (Tables 3 and 4). Interaction refers to the extent to which the joint effect of two risk factors on lung cancer differs from the independent effects of each of the factors. Two risk factors (*MTHFR *genotype and smoking/drinking) may act independently or interact thereby increasing or decreasing the effect of one another. A gene-environment interaction was suggested, with a combination of the TT genotype and smoking conferring significantly higher risk (OR = 6.16, 95% CI = 3.48 - 10.9), compared with at least one C allele and no history of smoking. Smoking (OR = 3.17) and the TT genotype of the C677T polymorphism (OR = 2.00) act independently (3.17*2.00 = 6.34 ≈ 6.16). There was no significant interaction between smoking and the C677T polymorphism with lung cancer. There was also no significant effect modification by the A1298C polymorphism in the association of smoking and lung cancer (P_interaction _= 0.11). However, the risk estimates indicated that smoking and the "at-risk" genotype did not seem to be associated with increased risk among those already at increased risk due to one of the two factors (smoking/"at-risk" genotype). Eight studies examined the interaction between smoking and the C677T or A1298C polymorphism [25,31-35,38,51]. Differences in the direction and the strength of the interaction were observed. The difference in circulating folate levels between different populations may partly account for the findings [29,52]. Furthermore, the differences may indicate a difference in susceptibility among different populations to smoking-induced lung cancer.

We evaluated the interaction between the C677T or A1298C polymorphism and alcohol drinking (Tables 3 and 4). Drinking (OR = 1.76) and the TT genotype of the C677T polymorphism (OR = 2.00) act independently (1.76*2.00 = 3.52 ≈ 3.09). No interaction of drinking and the C677T polymorphism with lung cancer was detected while we found a significant interaction between drinking and the A1298C polymorphism (P_interaction _= 0.049). Namely, excessive drinking (OR = 1.76) with the CC genotype (OR = 1.57) had an unexpectedly increased risk of lung cancer (1.76 *1.57 = 2.76 «7.23). As alcohol interferes with folate absorption and usage [53,54] and serves as a methyl group antagonist [55], it is biologically plausible. Four studies examined the interaction between drinking and the C677T polymorphism [25,29,33,36] or A1298C polymorphism [25,33]. Statistically significant interaction between the A1298C polymorphism and alcohol consumption was observed in women (P_interaction _= 0.021) but not in men (P_interaction _= 0.569) [33]. Similarly, a significant interaction between drinking habits and the A1298C genotype was found (P_interaction _= 0.025) but the CC genotype of the A1298C polymorphism was associated with a lower lung cancer risk (OR = 0.36, 95% CI = 0.12 - 1.04) [25]. Findings from gene-environment interaction analyses must be interpreted with caution due to reduced numbers of observations in the subgroups. Replication of findings is very important before any causal inference can be drawn. Testing replication in different populations is an important step.

Several limitations of this study warrant mention. Our study may have included a bias due to the self-reporting of smoking habits and alcohol consumption (misclassification bias). However, discrepancies between self-reported smoking habits and biochemical verification are minimal among the general population [56,57]. Similarly, the validity of self-reports on alcohol consumption is generally high [58,59]. Recall bias is one of well-recognized potential problems in case-control studies. Lung cancer patients may remember their exposure with a higher (lower) accuracy or completeness than healthy controls do (recall bias). To use hospital control is one way of minimizing the impact of recall bias because the controls would generally have the same incentive as the cases to remember events in the past [60]. Case-control studies tend to susceptible to selection bias, particularly in the control group. Controls should represent the source population from which the cases were drawn. As lung cancer patients are more likely to be more male sex and be less educated compared to controls [61], our results on sex ratio and educational status might not always suggest the existence of selection bias. When using hospital-based cases, it may not be possible to define the population which the cases were drawn. Hospital controls may be more appropriate because the study population can be defined as potential hospital users [60]. Generally, the reported participation rates were slightly higher in hospital-based case-control studies than in population-based case-control studies [62]. Participation rates of cases and controls were very high in this study. High participation rate may reduce the possibility of selection bias [62]. However, as the possibility of recall and selection biases could not be completely excluded in case-control studies, our findings should be interpreted with caution. Finally, we did not have data on dietary folate intake. Since smoking is associated with decreased circulating folate levels due to low folate intake [63,64], the observed interaction between alcohol consumption and the A1298 C polymorphism may be distorted by residual confounding by smoking. Additional studies are warranted to replicate our and others' findings from case-control genetic association studies.

## Conclusions

The TT genotype of the C677T polymorphism was significantly associated with an increased risk of lung cancer. Although the A1298C polymorphism was not associated with lung cancer, a significant interaction with drinking was observed. Smoking interacted with neither the C677T nor A1298C polymorphism in the development of lung cancer. Our results should be interpreted cautiously because we did not have data on dietary folate intake. Future studies involving larger control and case populations and better exposure histories will undoubtedly lead to a more thorough understanding of the role of MTHFR in lung cancer development.

## List of abbreviations used

CI: confidence interval; HWE: Hardy-Weinberg equilibrium; THF: tetrahydrofolate; MTHFR: methylenetetrahydrofolate reductase; OR: odds ratio; RFLP: fragment length polymorphism; SNP: single nucleotide polymorphism

## Competing interests

The authors declare that they have no competing interests.

## Authors' contributions

CK contributed to study design, laboratory work, data collection, data management, statistical analysis, data interpretation, and manuscript writing. TH, KT and YN participated in its design and coordination and helped to draft the manuscript. All authors read and approved the final manuscript.

## Pre-publication history

The pre-publication history for this paper can be accessed here:

http://www.biomedcentral.com/1471-2407/11/459/prepub
